# The importance of IFNα2A (Roferon-A) in HSV-1 latency and T cell exhaustion in ocularly infected mice

**DOI:** 10.1371/journal.ppat.1012612

**Published:** 2024-10-01

**Authors:** Shaohui Wang, Ujjaldeep Jaggi, Makoto Katsumata, Homayon Ghiasi

**Affiliations:** 1 Center for Neurobiology & Vaccine Development, Ophthalmology Research, Department of Surgery, Cedars-Sinai Medical Center, Los Angeles, California, United States of America; 2 Rodent genetics core facility, Department of Comparative Medicine, Cedars-Sinai Medical Center, Los Angeles, California, United States of America; University of Illinois at Chicago, UNITED STATES OF AMERICA

## Abstract

Published studies have generated compelling results indicating that type I IFN modulates function of HSV-1 latency-associated transcript (LAT). One member of type I IFN is IFNα2A also called Roferon-A). IFNα2A has been used in monotherapy or in combination therapy with other drugs to treat viral infections and different kinds of cancer in humans. The goal of this study was to determine whether the absence of IFNα2A affects primary and latent infections in ocularly infected mice. Therefore, we generated a mouse strain lacking IFNα2A expression (IFNα2A^-/-^). Ocular HSV-1 replication, IFN and immune cell expressions on days 3 and 5 post infection (PI), as well as eye disease, survival, latency-reactivation, and T cell exhaustion were evaluated in ocularly infected IFNα2A^-/-^ and wild type (WT) control mice. Absence of IFNα2A did not affect other members of the IFNα family but it affected IFNβ and IFNγ expressions as well as some immune cells on day 5 PI compared to WT mice. Viral replication in the eye, eye disease, and survival amongst ocularly infected IFNα2A^-/-^ mice were similar to that of WT infected mice. The absence of IFNα2A significantly reduced the levels of latency and T cell exhaustion but not time of reactivation compared with control mice. Our results suggest that blocking IFNα2A expression may be a useful tool in reducing latency and the subsequent side effects associated with higher levels of latency.

## Author summary

Many viruses have evolved strategies to circumvent the IFN system, including (*i*) inhibition of IFN production; (*ii*) blocking of downstream signaling events that occur after cytokine-receptor binding; and (*iii*) inhibition of the functions of proteins, which are induced by IFN, including apoptotic proteins. The interferons (IFNs) are of considerable interest in the control of HSV-1 infection due to their rapid generation in response to viral infection and their antiviral activities. Type I interferons, which includes interferon alpha (IFNα), are a family of cytokines important for control of viral infections. There are at least 14 IFNα genes in mouse, amongst which includes the gene encoding IFNα2A that play an important role in human studies. The mechanisms by which HSV-1 interferes with type I IFN responses during primary infection, establishment of latency and reactivation are not fully understood. Thus, to evaluate the importance of IFNα2A on HSV-1 infectivity, we generated IFNα2A knockout mice. The absence of IFNα2A significantly reduced latency and T cell exhaustion, but not virus reactivation.

## Introduction

In most pathogenic viral infections, the host responds immediately by producing type I interferons (IFNs) that limits viral replication, induces apoptosis/autophagy of infected cells, and contributes to mobilization of other immune responses [1,2]. Many viruses have evolved strategies to circumvent the IFN system [3], including (*i*) blocking of downstream signaling events that occur after cytokine-receptor binding; and (*ii*) inhibition of the functions of proteins, which are induced by IFNs, including apoptotic proteins [4]. IFNs are divided into three classes: type I, II and III [5]. Type I IFNs are powerful antiviral response factors capable of controlling viral infections and are essential for antiviral immunity in both mice and humans [6,7]. As IFN response also contributes to the development of the host immune response, defective IFN responses can result in skewed and suboptimal adaptive immune responses [8]. The type I IFN gene family in both human and mice are composed of at least 20 members: the IFNα subtypes, IFNβ, IFN*ω*, IFNε, and IFNκ [9–13]. Type I IFNs are immunoregulatory cytokines that have antiviral and antitumor activities [14–16]. IFNα/β have been studied extensively in the context of viral infections and have three major functions [5,17–21]: (**1**) Induction of antimicrobial responses; (**2**) Modulation of innate immune responses; and (**3**) Activation of the adaptive components of host antiviral defense. In addition, IFNα/β signaling can activate cell death pathways [22,23]. In most tissues, death of virally infected cells is a desired mode of virus containment [24]; however, the vertebrate host cannot tolerate death of neurons [25]. There are at least 14 IFNα genes that encode over 30 proteins and a single IFNβ gene in humans and mice [9,10]. All type I IFNs initiate their biological effects by binding to the same receptor, a heterodimer composed of the transmembrane proteins IFNAR1 and IFNAR2 [26,27]. IFNAR1^-/-^ mice (also known as IFNαβR^-/-^ or CD118^-/-^) lack antiproliferative and antiviral responses associated with IFNα/β signaling and are highly susceptible to viral infections [28,29] (Preliminary studies). Although the IFNα subtypes and IFNβ uses the same IFNARs, they exhibit functional differences [30].

Previous studies [31–34] have generated compelling results indicating that the latency-associated transcript (LAT) of HSV-1 modulates the effects of type I IFN induction in the trigeminal ganglia (TG) of wild-type (WT) mice after ocular infection with LAT(+) virus as compared with LAT(-) virus, which suggests that LAT acts to suppress the production of IFNα. Additionally, we have shown that LAT downregulates JAK1 and JAK2 pathways as well as affecting expressions of ISG-15, ISG-54, ISG-56, IP-10, OAS-3, IRF-1, IRF-3, Mx-1, CIITA, and GBP-1 genes but not TLR genes in latently infected mouse TG [32]. LAT has anti-apoptotic activity [35–40]. Replacing LAT with two different anti-apoptosis genes restores the WT reactivation phenotype to a LAT-minus virus [41,42] indicating that LAT’s anti-apoptosis activity is important for LAT reactivation phenotype. Thus, LAT functions at multiple levels.

In our model of ocular HSV-1 infection, LAT had no effect on the levels of NF-*κ*B expression in the TG of infected WT mice [32]. One member of IFNα family is IFNα2 and it has three isoforms of IFNα2A, IFNα2B, and IFNα2C [9]. IFNα2A also called Roferon-A is well characterized, broadly expressed with enhanced antiviral activities [9,43]. IFNα2A has been used in monotherapy or in combination therapy with other drugs to treat viral infections, including chronic hepatitis B and chronic hepatitis C, in addition to different kinds of cancer (including melanoma, renal cell carcinoma, leukemia, melanoma, AIDS-related Kaposi’s sarcoma), HIV, SIV, and LCMV [15,43–50]. However, the efficacy of IFNα2A therapy against viral infections and cancers was dependent on the time and dosage of treatment [51,52]. Overall, in both viral infection and cancer models, IFNα2A therapy showed greatest efficacy against early viral infections and cancer establishment [15].

The mechanisms by which HSV-1 interferes with type I IFN responses during primary infection, establishment of latency, and reactivation are not fully understood. Therefore, due to the prevalence of IFNα2A expression during viral infection and its use therapeutically for the treatment of malignancy and viral infections, we decided to characterize its functions during HSV-1 infection by constructing an IFNα2A knockout mice (IFNα2A^-/-^). In the present study, we investigated the impacts of the absence of IFNα2A expression on HSV-1 infectivity using IFNα2A^-/-^ mice. We show that in the absence of IFNα2A expression: 1) Viral replication was similar in the eyes of infected IFNα2A^-/-^ to WT control mice; 2) gB transcript in isolated TG of infected IFNα2A^-/-^ mice on day 3 was similar to control mice, but was significantly reduced in TG of infected IFNα2A^-/-^ mice on day 5 post infection; 3) On day 3 PI, except for IFNκ, no other significant differences were detected for IFNα1, IFNα6, IFNα11, IFNα13, and IFNα16 expressions in TG of IFNα2A^-/-^ mice compared with control mice, while on day 5 PI, expressions of IFNα1, and IFNα11 in TG of IFNα2A^-/-^ mice were significantly lower than in control mice; 4) absence of IFNα2A did not affect expressions of CD4, CD8α, F4/80, CD11b, CD11c, NK1.1, IRF9, Stat1, Stat2, IFNβ, or IFNγ in TG of infected mice on day 3 PI but its absence affected CD8α, F4/80, CD11c, NK1.1, IRF9, Stat1, Stat2, IFNβ, or IFNγ expressions on day 5 PI; 5) Levels of LAT RNA in TG of IFNα2A^-/-^ mice was significantly less than in control mice, while its absence did not affect reactivation time or eye disease; and 6) CD8α T cell exhaustion in TG of latently infected IFNα2A^-/-^ mice was significantly less than in control mice. The absence of IFNα2A in infected mouse correlated with lower latency and lower T cell exhaustion. Therefore, our results suggest that abolishing IFNα2A expression may present an effective way to reduce latency and pathology associated with higher LAT expression.

## Results

### Generation of IFNα2A mice

**Fig 1:**
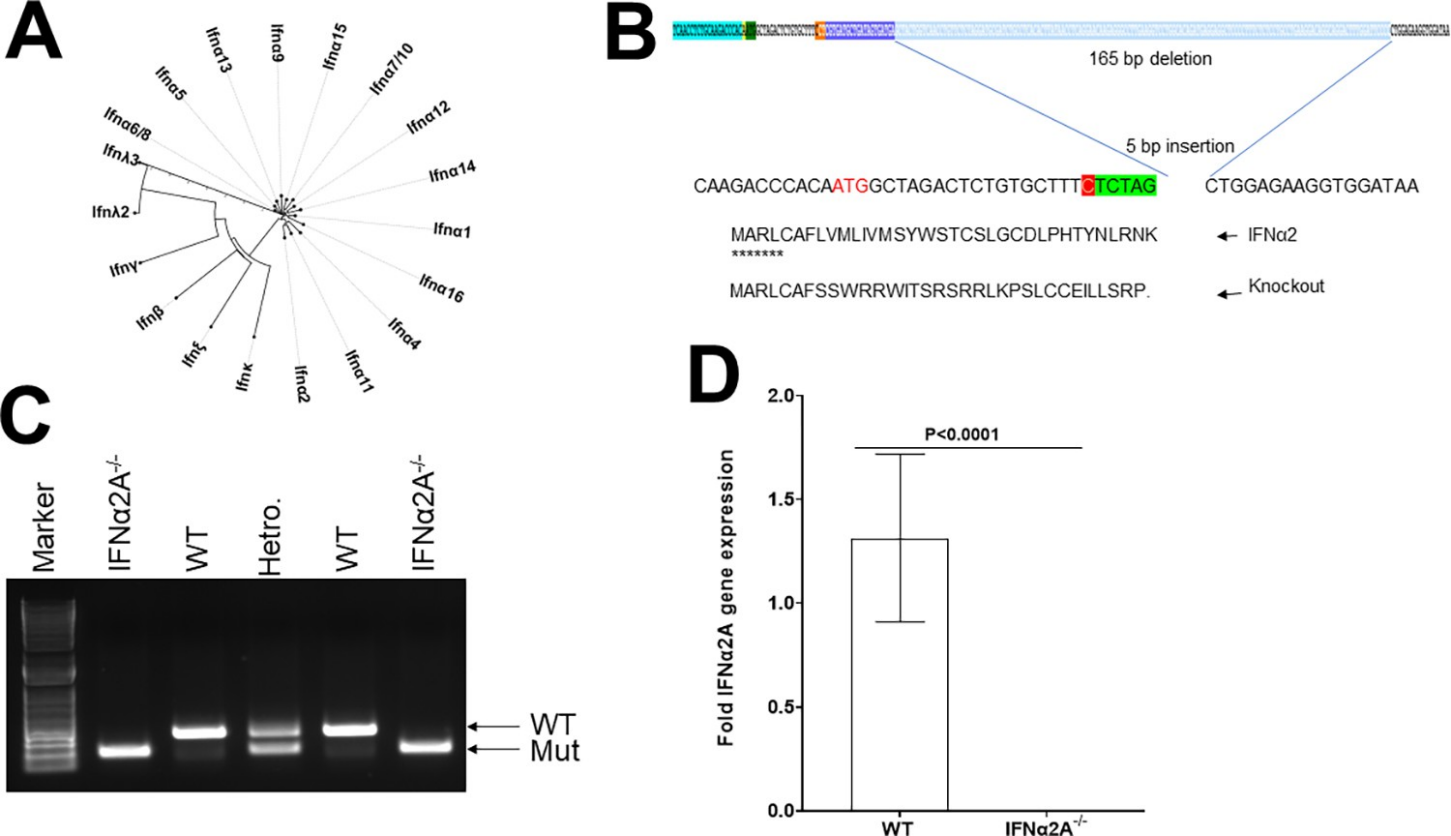
Generation of IFNα2A knockout mice. A) Phylogenetic tree of mouse interferons. Sequences of mouse interferons were aligned, and the phylogenetic tree was drawn by Clustal Omega, as described. As shown in Fig 1, there is a huge similarity between interferon alpha subtypes versus other interferons, the other type I interferon IFNβ, IFNε, and IFNκ are distant from alpha subtypes on the evolutionary tree: B) A schematic view of establishing IFNα2A deletion mice. The guide RNA sequence is CCUCGUGAUGCUGAUAGUGAUGA. The CLISPR resulted in a 165 bp deletion after 7th codon followed by 5 bp “TCTAG’ insertion, created a missense mutation and prematurely terminated at 34th amino acid; C) Representative genotyping of IFNα2A^-/-^ mice. As shown, the WT allele size is about 341 bp and the IFNα2A deletion allele size is 181 bp; and D) Absence of IFNα2A expression in TG of IFNα2A^-/-^ mice. TG from WT mice and IFNα2A knockout mice were harvested, expression of IFNα2A was measured by RT-qPCR as indicated. Each point represents mean ± SEM from 10 TG/group. Differences were detected using t-test.

Type I IFNs are divided into IFNα and IFNβ subtypes [14–16]. There is a single gene associated with IFNβ expression and IFNβ^-/-^ mice have been developed and studied extensively [53,54]. In contrast to IFNβ, there are at least 14 mouse IFNα genes (i.e., IFNα1, IFNα2, IFNα4, IFNα5, IFNα6, IFNα7, IFNα9, IFNα11, IFNα12, IFNα13, IFNα14, IFNα15, IFNα16, and IFNαB) [9,10]. Among the 14 known IFNα genes (Fig 1A), IFNα2A, also called IFNα2 or Roferon-A, is well characterized and has been used in the treatment of viral infections and different kinds of cancers [15,43–50]. However, there are no IFNα2A knockout mice available and given its extensive use in human studies, we generated IFNα2A knockout mouse lines with disabled IFNα2A allele with various degrees of indels using the CRISPR/Cas9 genome editing technology [55]. We utilized the CRISPR algorithm to design the guide RNA sequence [56] of UCAUCACUAUCAGCA UCACGAGG (20 bases plus PAM). We performed pronuclear microinjection of the CRISPR RNP in combination with a synthesized tracrRNA (Dharmacon U-002005-5) and wild type Cas9 protein (ESPCAS9 PRO-50UG, Millipore Sigma) at the concentration of 60 ng/μl crRNA/tracrRNA (approx. 1:1 molar ratio) mix and 50 ng/μl Cas9 protein. We observed different degrees of insertion or large deletions in 4 founders and chose one of the founders (founder #3) carrying a 5 bp insertion followed by 165 bp deletion that creates a missense mutation after 7^th^ amino acid and premature termination after 34^th^ amino acid (Fig 1B). Deletion was confirmed with sequencing. Mice having this deletion were back crossed and the absence of deletion was confirmed by PCR (Fig 1C). We used qRT-PCR to compare IFNα2A RNA expression in IFNα2A^-/-^ and control mice. RNA levels in isolated TG from IFNα2A^-/-^ mice were not detectable (Fig 1D). Thus, IFNα2A^-/-^ mice lacked expression of IFNα2A. This strain of knockout mouse grew and bred normally, and no anatomical dysfunction was detected in these mice.

### Viral replication in the eyes of ocularly infected IFNα2A^-/-^ mice was not affected by the absence of IFNα2A expression

**Fig 2:**
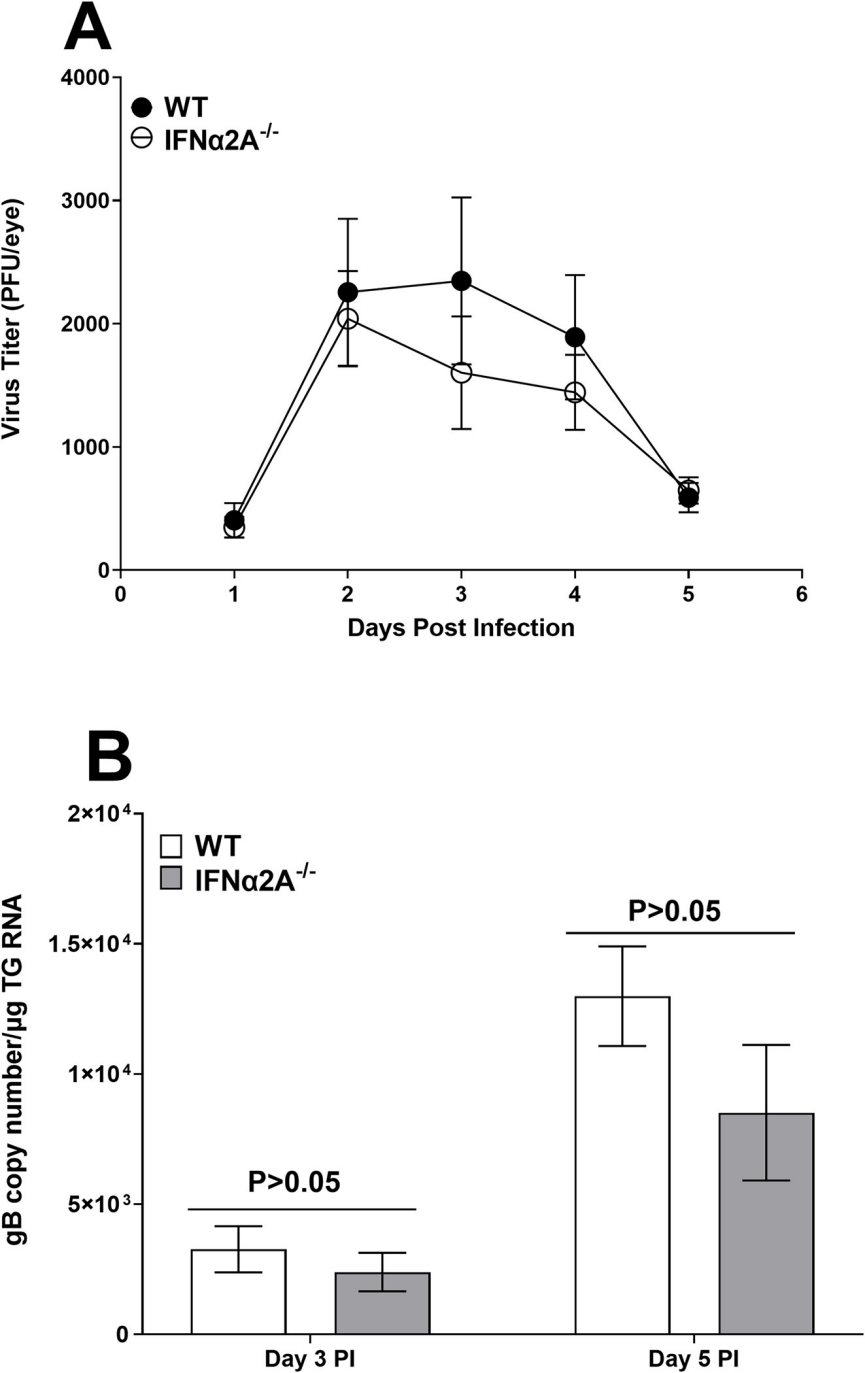
Virus titers and gB expression in the eyes and TG of infected mice. (A) Virus titers in the eyes of IFNα2A^-/-^ mice. IFNα2A^-/-^ and control mice were ocularly infected with 2 X 10^5^ PFU/eye of HSV-1 strain McKrae. Tear films were collected on days 1 to 5, and virus titers were determined by standard plaque assays. Each point represents the mean titer of 20 eyes for WT and 34 eyes for IFNα2A^-/-^ mice from two separate experiments; and (B) HSV-1 gB expression in TG of infected mice. IFNα2A^-/-^ and control mice were ocularly infected with 2 X 10^5^ PFU/eye of HSV-1 strain McKrae. TG were harvested on days 3 and 5 PI. Total RNA was extracted and gB copy number was measured by RT-qPCR and normalized with GAPDH. Each point represents the mean ± SEM gB copy numbers of 12 TG for day 3 and 10 TG for day 5 for each group from two separate experiments. Differences were detected using t-test.

To determine if the absence of IFNα2A expression alters viral replication in the eyes of infected mice, IFNα2A^-/-^ and control mice were ocularly infected with HSV-1 strain McKrae. Tear films were collected from 20 eyes of WT and 34 eyes of IFNα2A^-/-^ mice in two separate experiments on days 1–5 PI and the presence of infectious virus was determined by standard plaque assays (Fig 2A). Virus titers in the eyes of the two infected mice groups were similar on days 1 to 5 PI (Fig 2A, p>0.05 at all-time points). Thus, the absence of IFNα2A in IFNα2A^-/-^ mice was not associated with reduced viral replication in the eyes of infected mice.

Fig 2A showed that the absence of IFNα2A did not affect viral replication in the eyes of infected mice. We next asked whether the lack of IFNα2A affected expression levels of HSV-1 gB transcript in TG of infected mice on days 3 and 5 PI. IFNα2A^-/-^ and control mice were infected as above with HSV-1 McKrae. On days 3 and 5 PI, TG from infected mice were collected and total RNA was isolated from each TG. RNA isolated from each TG was analyzed by TaqMan RT-PCR to determine copy numbers for gB mRNA. GAPDH mRNA in each sample was used as an internal control. The results showed lower levels of each transcript in TG of infected IFNα2A^-/-^ mice than in control mice on both days 3 and 5 PI. While the differences were not statistically significant on day 3 PI, they were significant on day 5 post infection (Fig 2B; p>0.05 for day 3 and p<0.05 on day 5 PI). Collectively, these results indicate that, while the differences between infected WT and IFNα2A mice were not statistically significant for virus replication and gB copy numbers during primary infection, yet a trend for decreased replication that fits with decreased copies of gB transcript in the corneas and TG of IFNα2A infected mice compared with WT control mice were detected.

### Expressions of various cellular transcripts in TG of IFNα2A^-/-^ mice during primary ocular infection

**Fig 3:**
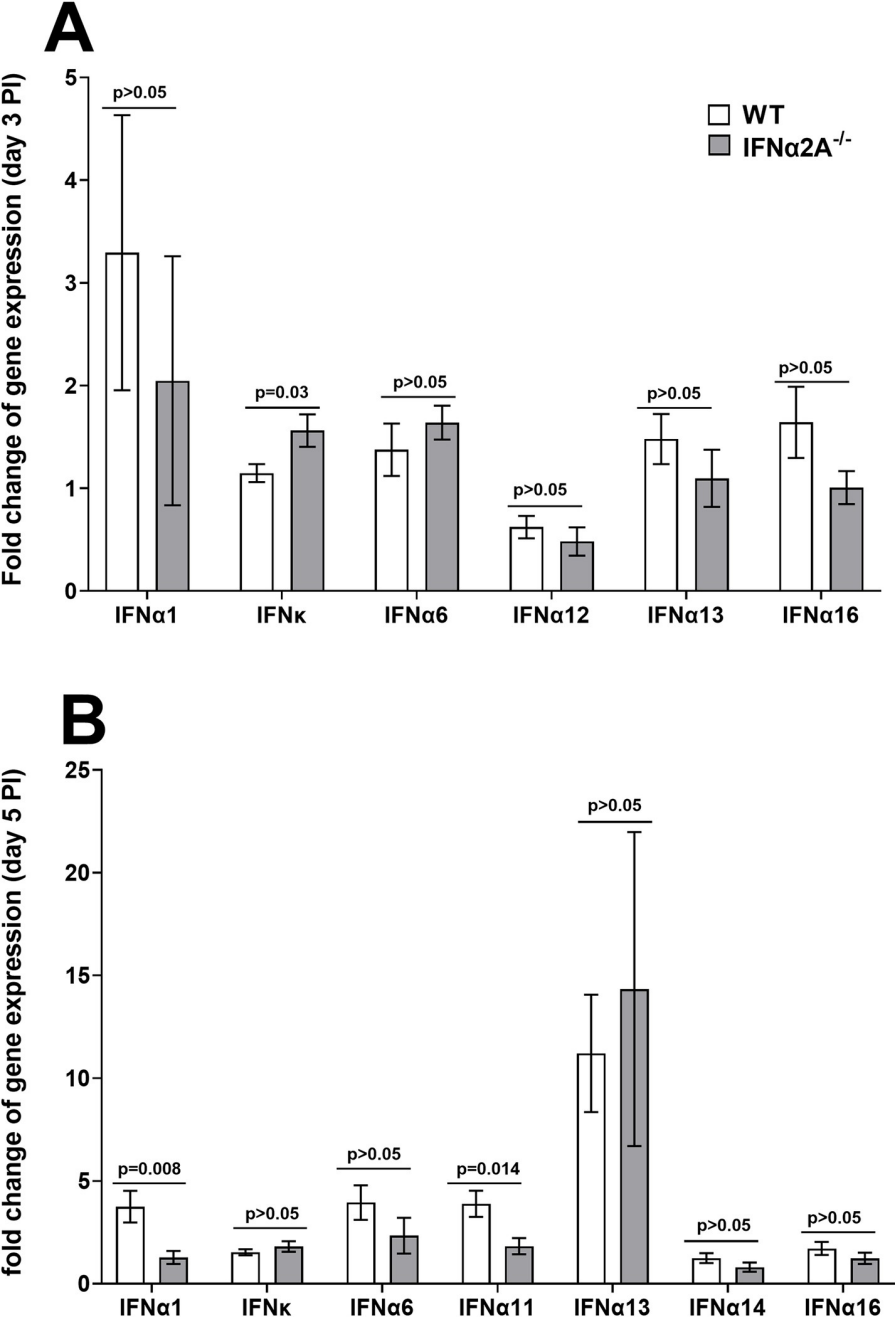
Absence of IFNα2A and its effects on expressions of IFNα family members during primary infection. IFNα2A^-/-^ and WT mice were infected ocularly with 2x10^5^ PFU/eye of McKrae virus. On days 3 and 5 PI, TG from infected mice were isolated and qRT-PCR was performed using specific TaqMan primers. TG from uninfected mice was used for normalization and GAPDH was used as the endogenous control. Results are shown as mean ± SEM from two separate experiments using 12 TG for day 3 and 10 TG for day 5 for each group. Differences were detected using t-test.

Fig 2 above showed that the absence of IFNα2A did not affect viral replication in the eyes of infected mice but reduced the gB expression in TG of infected mice on day 5 PI. We next asked whether the lack of IFNα2A altered some of the other members of the IFNα family. IFNα2A^-/-^ and control mice were infected as above. On days 3 and 5 PI, TG from infected mice were collected and RNA was isolated and expressions of IFNα1, IFNκ, IFNα6, IFNα11, IFNα13, IFNα14, and IFNα16 mRNAs were determined by TaqMan RT-PCR. Results are presented as “fold” increase over baseline mRNA levels in TG of uninfected IFNα2A^-/-^ and WT mice for each group. GAPDH mRNA in each sample was used as an internal control. The results showed no significant differences in the levels of each transcript in TG of infected IFNα2A^-/-^ mice than in control mice except for IFNκ on day 3 PI (Fig 3A). In contrast to day 3 PI, levels of IFNκ expression on day 5 PI were not significantly different, while levels of IFNα1 and IFNα11 transcripts were significantly lower in IFNα2A^-/-^ mice than in control mice (Fig 3B). Collectively, these results indicate that except for a few IFNα genes, the absence of IFNα2A in TG of infected mice did not alter expressions of many members of the IFNα family compared with WT infected mice.

**Fig 4:**
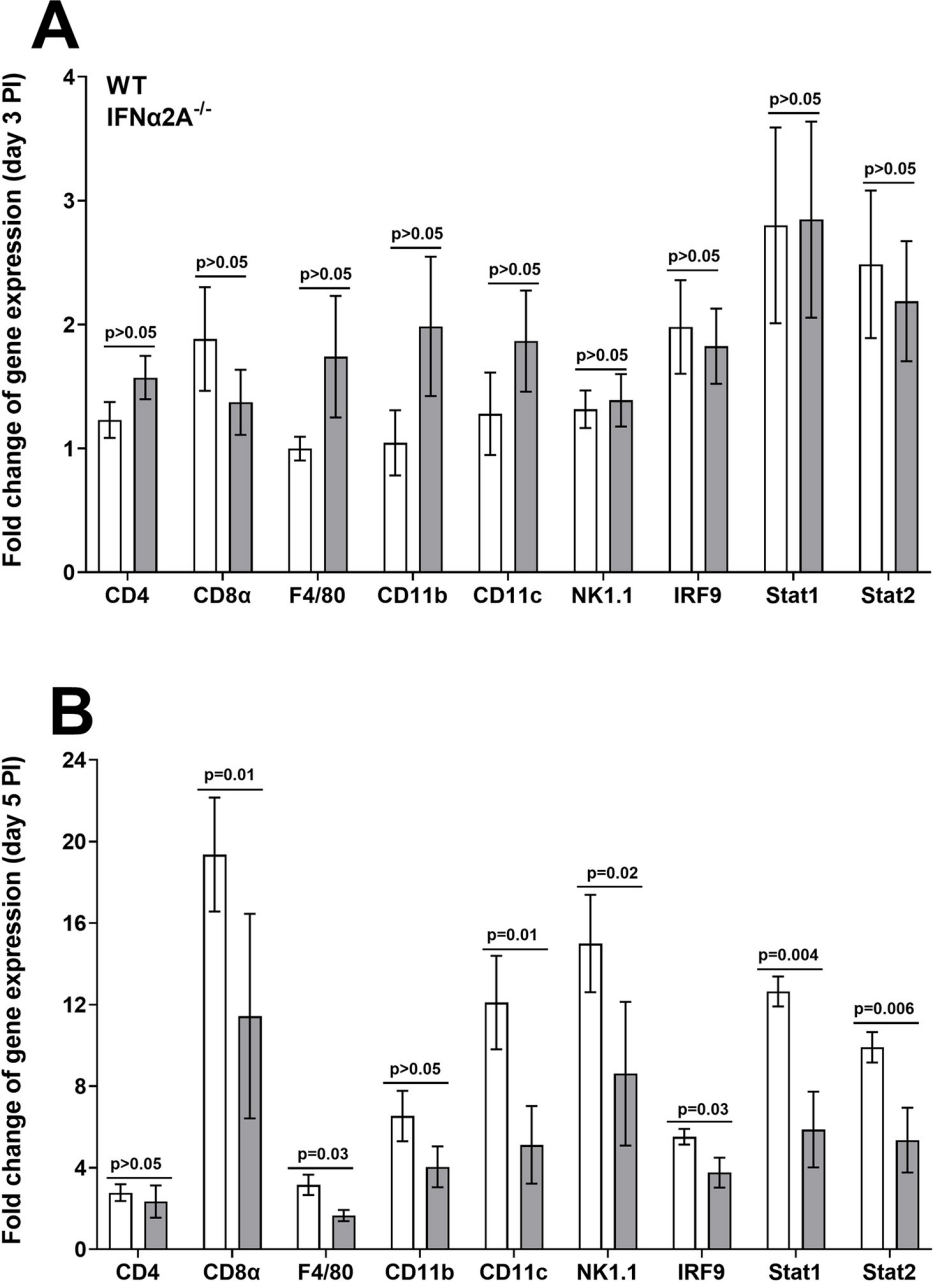
Immune cell profiling in the absence of IFNα2A during primary infection. RNA isolated on days 3 and 5 PI from IFNα2A^-/-^ and WT infected mice were used to measure the expression levels of CD4, CD8α, F4/80, CD11b, CD11c, Nk1.1, IRF9, Stat1 and Stat2. qRT-PCR were done as above and GAPDH was used as an endogenous control. TG from IFNα2A^-/-^ and WT uninfected mice were used for normalization. Each point represents the mean ± SEM fold change of expressions of 12 TG for day 3 and 10 TG for day 5 for each group from two separate experiments. Differences were detected using t-test.

The above results suggest that the absence of IFNα2A does not significantly affect the expressions of several members of the IFNα family. Therefore, we next asked whether the absence of IFNα2A in IFNα2A^-/-^ mice affects the expressions of CD4, CD8α, F4/80, CD11b, CD11c, NK1.1, IRF9, Stat1, and Stat2 transcripts in TG of infected mice on days 3 and 5 PI. RNA isolated from IFNα2A^-/-^ and control mice (see Fig 3) was used to measure CD4, CD8α, F4/80, CD11b, CD11c, NK1.1, IRF9, Stat1, and Stat2 transcript levels in TG of infected mice by qRT-PCR. Results are presented as “fold” increase over baseline mRNA levels in TG of naive mice for each group. GAPDH mRNA in each sample was used as an internal control. On day 3 PI, CD4, CD8α, F4/80, CD11b, CD11c, NK1.1, IRF9, Stat1, and Stat2 transcripts were similar in mice with and without IFNα2A expression (Fig 4A, p>0.05). On day 5 PI, CD8α (Fig 4B; p = 0.01), F4/80 (Fig 4B; p = 0.03), CD11c (Fig 4B; p = 0.01), NK1.1 (Fig 4B; p = 0.02), IRF9 (Fig 4B; p = 0.03), Stat1 (Fig 4B; p = 0.004), and Stat2 (Fig 4B; p = 0.006) transcript levels were significantly reduced in TG of IFNα2A^-/-^ mice compared with control mice. However, the levels of CD4 and CD11b transcripts were not significantly different in IFNα2A^-/-^ mice compared with control mice (Fig 4B). These results suggest that absence of IFNα2A significantly reduced all tested immune related genes except CD4 and CD11b by day 5 PI.

**Fig 5:**
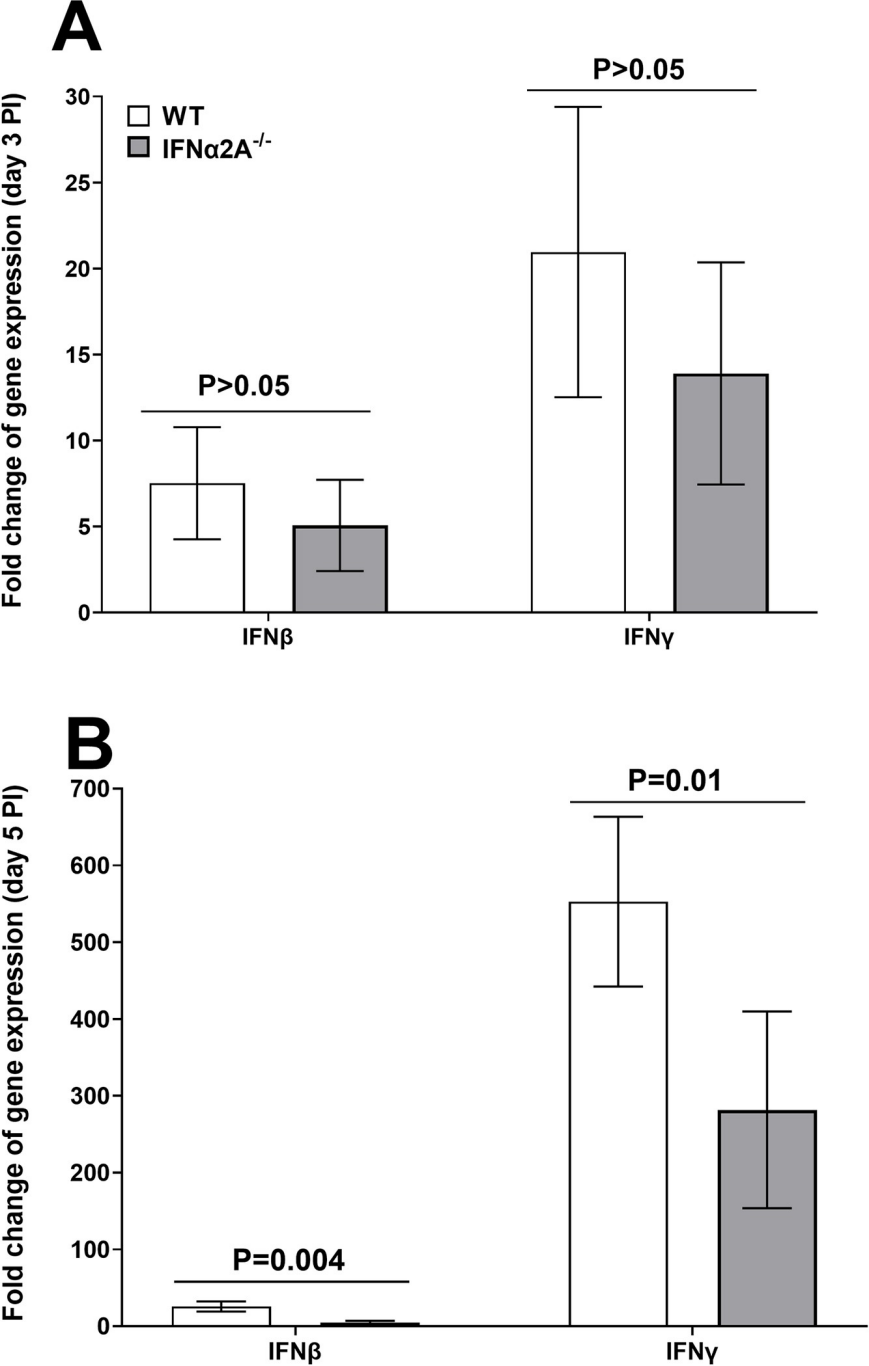
Effects of the absence of IFNα2A on IFNβ and IFNγ expression during primary infection. RNA isolated on days 3 and 5 PI from IFNα2A^-/-^ and WT infected mice were used to measure the expression levels of IFNβ and IFNγ. qRT-PCR were done as above and GAPDH was used as the endogenous control. TG from uninfected mice was used for normalization. Each point represents the mean ± SEM fold change of gene expression two separate experiments using 12 TG for day 3 and 10 TG for day 5 for each group. Differences were detected using t-test.

Our results described in Fig 4 suggested that, in the absence of IFNα2A by day 5 PI, expressions of the tested immune response genes were significantly reduced in TG of infected mice. Thus, we next asked whether the absence of IFNα2A in IFNα2A^-/-^ mice affects expression levels of IFNβ and IFNγ transcripts in TG of infected mice on days 3 and 5 PI. IFNβ is a member of type I IFN family [54], while IFNγ is type II IFN consists of a single gene [17]. It has been suggested that IFNβ and IFNγ play opposing roles in the regulation of inflammatory responses: IFNγ promotes inflammatory responses, whereas IFNβ has anti-inflammatory properties [57–59]. RNA isolated from IFNα2A^-/-^ and control mice (see Fig 3, above) was used to measure IFNβ and IFNγ transcripts levels in TG of infected mice by qRT-PCR. Results are presented as “fold” increase over baseline mRNA levels in TG of naive mice for each group. GAPDH mRNA in each sample was used as an internal control. On day 3 PI, IFNβ and IFNγ expression levels were similar in mice with and without IFNα2A expression (Fig 5A, p>0.05). In contrast to day 3, expression levels of IFNβ (Fig 5B; p = 0.004) and IFNγ (Fig 5B; p = 0.01) transcripts were significantly reduced in TG of IFNα2A^-/-^ mice compared with control mice on day 5 PI. These results suggest that the absence of IFNα2A significantly reduced IFNβ and IFNγ expressions by day 5 PI.

### Latency is reduced in the TG of latently infected mice lacking IFNα2A expression

**Fig 6:**
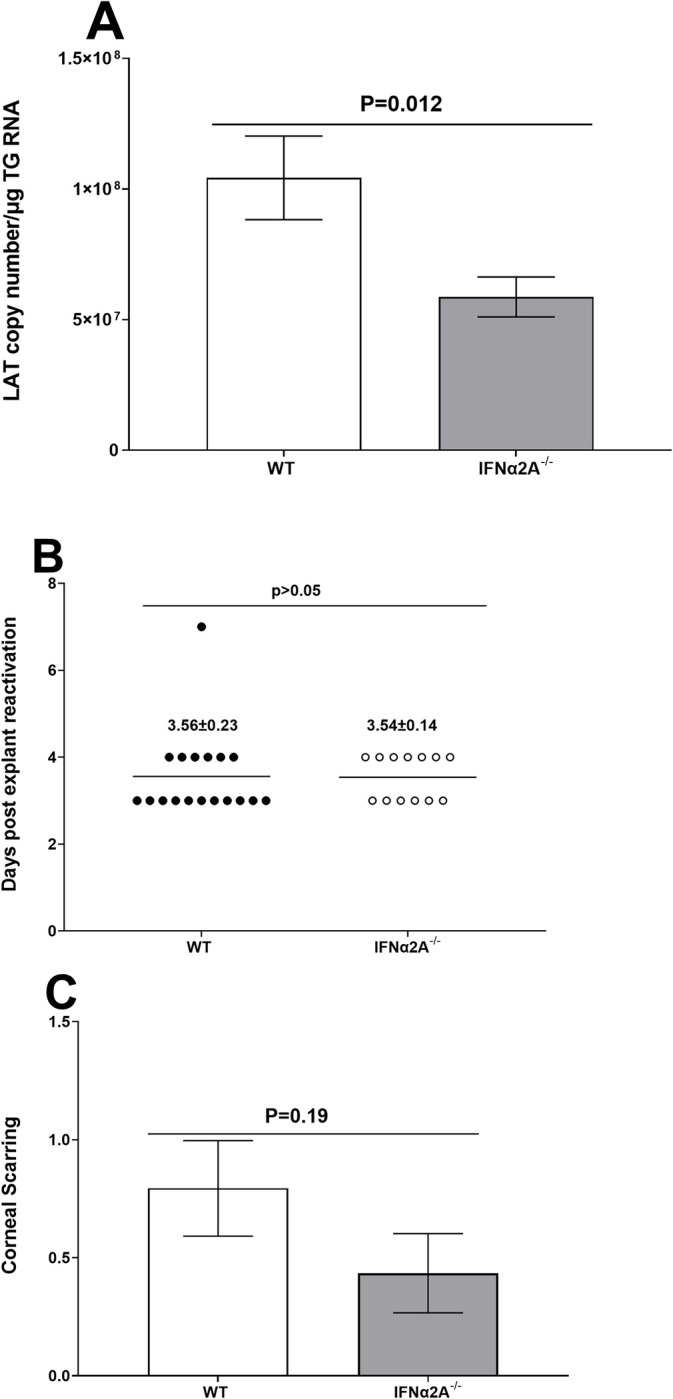
Role of IFNα2A in HSV-1 latency, reactivation, and corneal scarring. A) Levels of latency in TG of latently infected mice. IFNα2A^-/-^ and control mice were ocularly infected with 2 X 10^5^ PFU/eye of HSV-1 strain McKrae. TG were harvested on day 28 PI and total TG RNA was isolated as described in Materials and Methods. LAT RNA copy numbers were measured by qRT-PCR using a standard curve generated with pGem5317. GAPDH expression was used to normalize relative levels of LAT RNA expression. Each bar represents the mean ± SEM from 20 mice from three independent experiment; B) Virus reactivation in infected mice. IFNα2A^-/-^ and control mice were ocularly infected with 2 X 10^5^ PFU/eye of HSV-1 strain McKrae as above. TG from infected mice were harvested on day 28 PI and the explant reactivation assay was performed. Data points indicate the day of virus reactivation. Each point is the mean ± SEM from 18 TG for WT control mice and 13 TG for IFNα2A^-/-^ mice; and C) corneal scarring in infected mice. Severity of corneal scarring on mice used to measure latency-reactivation was determined on day 28 PI. Each point represents the mean ± SEM from 46 eyes for control mice and 38 eyes for IFNα2A^-/-^ mice. Differences were detected using t-test.

The above results suggests that levels of immune cells were significantly reduced in IFNα2A^-/-^ mice compared with control mice (Figs 4 and 5). To determine whether the absence of IFNα2A affects latency, IFNα2A^-/-^ mice and control mice were infected ocularly as above and TG from infected mice were isolated on day 28 PI and total TG RNA was isolated from latently infected TG. Isolated TG RNA was used to quantify LAT RNA copy numbers and cellular GAPDH RNA was used as an internal control. The amount of LAT RNA during latency in TG from IFNα2A^-/-^ mice was significantly reduced compared with WT mice (Fig 6A; p = 0.012). These results suggest that, in the absence of IFNα2A, latency in TG of HSV-1 infected mice was significantly reduced.

### Time of explant reactivation is not affected in latently infected mice lacking IFNα2A expression

The qRT-PCR analyses described in Fig 6A suggests that the absence of IFNα2A reduces LAT expression in TG of latently infected mice. Thus, we asked whether reduced LAT levels correlated with reduced reactivation of latent virus. To test this, IFNα2A^-/-^ or control mice were ocularly infected with 2 X 10^5^ PFU/eye of HSV-1 strain McKrae. On day 28 PI, individual TG from infected mice was harvested and the kinetics of virus reactivation was measured in explanted TG. Average reactivation time for IFNα2A^-/-^ mice was 3.56 ± 0.23 days; control mice reactivated similarly, with an average of 3.54 ± 0.12 days (Fig 6B, p>0.05). Together, these results suggest that absence of IFNα2A does not affect time to reactivation compared with control mice despite reduced levels of latency in IFNα2A^-/-^ mice. Thus, in the absence of IFNα2A time to reactivation is not correlated with the levels of latency.

### Absence of IFNα2A does not affect levels of corneal scarring or mice survival

A total of 47 IFNα2A^-/-^ mice and 44 control mice (from 4 separate experiments) were ocularly infected with 2x10^5^ PFU/eye of HSV-1 strain McKrae as above. Survival of infected mice was recorded at day 28 PI. Forty-two of 47 (89%) of mice in the IFNα2A^-/-^ group and 40 of 44 (91%) in the control group survived ocular infection. No significant differences in survival rate were detected between the IFNα2A^-/-^ infected mice and control mice (p>0.05, Mann Whitney test) (not shown). Eyes of the surviving mice were examined for corneal scarring (CS) at day 28 PI. Although the IFNα2A^-/-^ infected mice had lower levels of CS compared with control mice, these differences were not statistically significant (Fig 6C; p = 0.19). Thus, the absence of IFNα2A did not exacerbate CS or result in increased mortality of infected mice.

### Absence of IFNα2A in IFNα2A^-/-^ mice significantly reduced CD8α T cell exhaustion markers during latency

**Fig 7:**
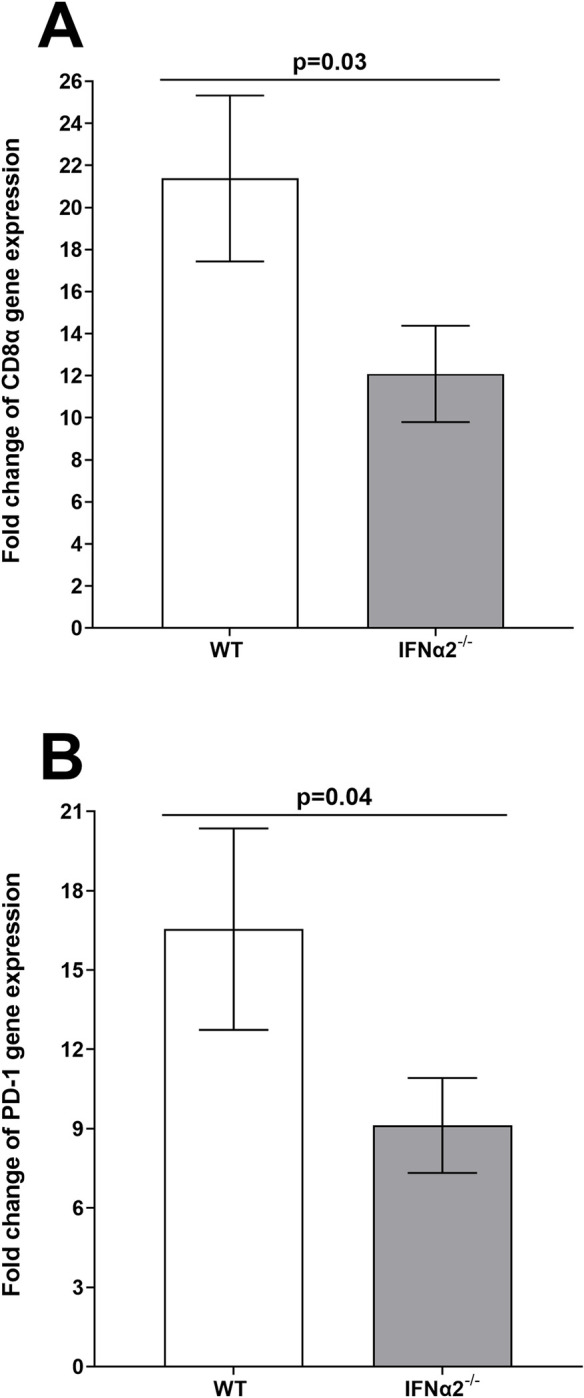
T cell exhaustion in the absence of IFNα2A in TG of latently infected mice. Isolated RNA from total TG (see Fig 6B) was used to measure expressions of CD8a (panel A) and PD-1 (panel B) transcripts by qRT-PCR. CD8α and PD-1 expression in TG of uninfected naive mice was used as a baseline to estimate relative expression of each transcript in TG of infected mice. GAPDH expression was used to normalize relative expressions of each transcript. Each bar represents mean ± SEM from 22 TG for control mice and 35 TG for IFNα2A^-/-^ mice from two independent experiments. Differences were detected using t-test.

The above results suggested that the absence of IFNα2A reduces LAT expression in TG of latently infected mice compared with WT control mice (Fig 6B). Previously, we have shown that reduced latency in TG of latently infected mice correlated with lower levels of CD8α and PD-1 expressions [60–62]. Thus, to investigate the effects of reduced LAT expression in TG of latently infected IFNα2A^-/-^ mice on T cell exhaustion, IFNα2A^-/-^ and WT control mice were infected as above with HSV-1 strain McKrae. The relative levels of CD8 and PD-1 were determined by RT-PCR of total TG extracts (Fig 7). Results are presented as “fold” increase (or decrease) compared to the baseline mRNA levels in TG from uninfected naive mice for each group. GAPDH mRNA in each sample was used as an internal control. Expression levels of CD8α transcript in TG of IFNα2A^-/-^ mice was significantly lower than in control mice (Fig 7A, p = 0.03). PD-1 transcript level was also significantly lower in TG of latently infected IFNα2A^-/-^ mice compared with control mice (Fig 7B, p = 0.04). These results suggest that absence of IFNα2A does affect expression levels of CD8α and PD-1 in TG of latently infected mice and this strongly correlates with lower levels of latency as we reported previously [60–62].

## Discussion

In most pathogenic viral infections, the host responds immediately by producing type I interferons (IFNs) that limits viral replication, induces apoptosis/autophagy of infected cells, and contributes to mobilization of other immune responses [1]. Many viruses have evolved strategies to circumvent the IFN system [3], including (*i*) inhibition of IFN production; (*ii*) blocking of downstream signaling events that occur after cytokine-receptor binding; and (*iii*) inhibition of the functions of proteins, which are induced by IFN, including apoptotic proteins [4]. IFNα/β acts in both autocrine and paracrine manner [63,64] and affects expressions of over 1,000 genes [65,66]. The mechanisms by which HSV-1 interferes with type I IFN responses during primary infection and in the establishment of latency and reactivation are not fully understood. Our published studies [31,32] and our analyses of ocular HSV-1 infection and recurrences have generated compelling results indicating that HSV-1 modulates the effects of type I IFN. Amongst the 14 IFNα genes, IFNα2A is well characterized and broadly expressed, and has been used in human studies [15,43–50]. In contrast to IFNα2A, the other 13 members of IFNα are not well characterized, are more tissue restricted, and are not represented in all mammalian species [54]. Since human studies have shown the importance of IFNα2A in virus and cancer therapy and there is no knockout of IFNα2A available to evaluate the role of IFNα2A in HSV-1 infectivity, we deleted this gene to generate IFNα2A knockout mice. The IFNα2A^-/-^ mice generated in this study allowed us to evaluate the role of IFNα2A during primary and latent HSV-1 infection. As expected, IFNα2A expression was not detectable in TG of IFNα2A^-/-^ mice compared with control mice and deletion of IFNα2A had no obvious side effects in mice.

IFNα/β is essential in the control of HSV-1 infection in the eyes of ocularly infected mice [67,68] and ectopic expressions of IFNα or IFNβ markedly suppresses HSV-1 replication, its spread and is associated with a reduction in lytic gene expression in infected mice [69,70]. Mice deficient in IFN responses exhibits lethal susceptibility to murine cytomegalovirus (MCMV) infection [71]. It is also reported that mutations within STAT1 which is an essential downstream factor in IFNα/β and IFNγ signaling pathways leads to lethal outcomes from infection with HSV-1, HCMV, and Epstein-Barr virus [72–75]. We also observed lower levels of IRF9, Stat1, and Stat2, which indicated a less robust interferon response via Jak/Stat signal pathways in the knockout mice. Recombinant IFNα has also been used to treat HCMV-induced retinitis after HIV infection [76]. Our current study focused on determining the roles of the absence of IFNα2A on HSV-1 infection. We found that the absence of IFNα2A did not affect virus replication in the eyes of infected IFNα2A^-/-^ mice compared with control mice, but it does reduce the HSV-1 gB expression in TG of infected mice on day 5 PI. We also looked at expressions of the remaining 13 members of IFNα and detected IFNα1, IFNκ, IFNα6, IFNα11, IFNα13, IFNα14, and IFNα16 mRNAs in TG of both IFNα2A^-/-^ and control mice, while levels of IFNα4, IFNα5, IFNα7, IFNα9, IFNα12, IFNα15, and IFNαB in TG were not detectable. Additionally, on day 3 PI, amongst the 6 detectable members of IFNαs in TG of IFNα2A^-/-^ mice compared with control mice, only IFNκ levels were upregulated in IFNα2A^-/-^ mice compared with control mice, however by day 5 PI, the differences were not significant. In contrast, levels of IFNα1 and IFNα11 were significantly reduced in IFNα2A^-/-^ mice compared with control mice at day 5 PI. IFNα11 is a part of the same cluster as IFNα2A and the absence of IFNα2A may affect expression of IFNα11 (Fig 1A). T cells, macrophages, DCs, NK cells play a major role in protection against HSV-1 infection [77–80]. During acute infection, innate lymphocytes such as neutrophils, NK cells, and macrophages are recruited to the site of infection to control the virus [81]. In this study, on day 3 PI, we did not detect any differences in the levels of immune infiltrates in TG of infected mice, while by day 5 PI, IFNα2A^-/-^ mice had significantly lower levels of CD8α, F4/80, CD11b, CD11c, NK1.1 compared with control mice. In conjunction with these reduced expressions of immune responsive genes, HSV-1 gB transcripts were also lower in the knockout mice, indicating reduced viral replication in TG on day 5 PI. Absence of differences in immune cells between IFNα2A^-/-^ mice and control mice on day 3 PI is possibly due to lower viral load in TG on day 3 versus day 5 PI. This lower viral load on day 3 versus day 5 PI correlated with significantly lower expression levels of CD8α, F4/80, CD11b, CD11c, NK1.1, IRF9, Stat1, and Stat2 transcripts. Similarly, levels of IFNβ and IFNγ in TG of infected IFNα2A^-/-^ and control mice were similar on day 3 PI, while the expression levels for both IFNβ and IFNγ were significantly lower in the IFNα2A^-/-^ group on day 5 PI. Thus, absence of IFNα2A significantly reduced levels of IFNβ and IFNγ expressions in TG of infected mice but the absence of IFNα2A expression did not affect viral replication in the eyes of infected mice. The absence of IFNα2A in infected mice did not affect their susceptibility to ocular infection with the virulent HSV-1 strain McKrae. Furthermore, after infection of mice lacking IFNα2A, the levels of eye disease were similar to that in control mice. Overall, lower expressions of CD8α, F4/80, CD11b, CD11c, NK1.1 IRF9, Stat1, Stat2, IFNβ, and IFNγ in TG did not affect virus replication in the eye, eye disease, or survival in IFNα2A^-/-^ infected mice. IFNAR1^-/-^ mice are highly susceptible to HSV-1 infection [82–85]. In contrast to the highly susceptible IFNAR1^-/-^ mice, IFNα2A^-/-^ mice are less susceptible to HSV-1 infection, which could be due to functional redundancy of IFNα subtypes.

HSV-1 LAT is the only viral product that is expressed abundantly in TG of infected mice, rabbits, and humans [60,86–89]. In this study we showed significantly less LAT expression in TG of IFNα2A^-/-^ mice than in control mice. LAT interferes with host IFNα/β expressions in the TG of infected mice and this interference is independent of other HSV-1 genes that inhibits IFNα/β signaling [90–95]. LAT is important for the high WT rate of *in vivo* spontaneous [96], and induced [86] reactivation from latency. LAT also has anti-apoptotic activity [35–40] and is associated with T cell exhaustion in TG of latently infected mice [60]. It was previously shown that LAT delays expressions of several IFNα subtypes as well as IFNβ *in vitro* and *in vivo* [34]. In addition to LAT, ICP0 and ICP27 also inhibits the type I IFN pathway during the acute phase of infection [97–99]. Our results suggest that IFNα2A could utilize a reverse mechanism on LAT expression to reduce levels of latency and T cell exhaustion during latency in mice. Similar to HSV-1 LAT, viruses have developed strategies to suppress IFNα/β such as vaccinia virus E3L and influenza virus NS1 that inhibits IFNα/β production [100–102].

Previously, we and others have shown that higher LAT expression is correlated with higher reactivation in infected mice [60,62]. HSV-1 LAT plays a major role in enhancing the reactivation phenotype in TG of infected mice and rabbits [86,96,103]. LAT deletion mutant viruses have a reduced reactivation phenotype in both mice and rabbits [86,96,103]. In this study, the absence of IFNα2A expression in TG of IFNα2A^-/-^ mice did not alter time of reactivation compared with control mice. However, levels of reactivation in this study were similar between latently infected IFNα2A^-/-^ and control mice despite significantly higher levels of LAT expression in the TG of control mice. Thus, reduced expression of IFNα2A transcript did not correlate with changes in time to reactivation. Consistent with reduced levels of LAT in TG of IFNα2A^-/-^ mice compared with control mice, we also found lower levels of CD8α and PD-1 expressions in TG of mice lacking IFNα2A. We previously showed that lower levels of latency strongly correlate with lower levels of CD8α and PD-1 expressions and, thereby, reduced T cell exhaustion [60]. Although despite the presence of other known IFNα genes, in this study we have shown that the absence of IFNα2A was not compensated by the other 13 IFNα subtypes with regards to HSV-1 latency and T cell exhaustion.

In conclusion, in this study, due to the importance of IFNα2A in both viral infections and cancer therapy, we report the novel construction of a knockout mouse lacking IFNα2A (Roferon-A) gene. The absence of IFNα2A affected levels of viral transcripts which leads to less cell recruitment and hence less inflammation during primary infection as well as reduced levels of latency and T cell exhaustion, but not time to reactivation. Our results also suggest that few but not all functions of IFNα2A could be compensated by the remaining members of the IFNα family and highlight the redundancy of the IFNα subtypes. Thus, blocking IFNα2A expression may be a useful tool to reduce latency and subsequently managing infection.

## Material and methods

### Ethics statement

All animal procedures were performed in strict accordance with the Association for Research in Vision and Ophthalmology Statement for the Use of Animals in Ophthalmic and Vision Research and the NIH guide for Care and Use of Laboratory Animals (ISBN 0-309-05377-3). The animal research protocol was approved by the Institutional Animal Care and Use Committee of Cedars-Sinai Medical Center (Protocol # 8837).

### Cells and virus

The rabbit skin (RS) cell line was cultured in minimal essential medium plus 5% fetal bovine serum and maintained as described previously [104]. Triple-plaque-purified HSV-1 strain McKrae was grown in RS cell monolayers as described previously [96]. HSV strain McKrae is a virulent strain of virus that infects mice efficiently without corneal scarification.

### Generation of IFNα2A knockout mice

IFNα2A^-/-^ mice on a C57BL/6 background were generated in-house using CRISPR technology. The gRNA sequence used was UCAUCACUAUCAGCA UCACGAGG and was synthesized by Dharmacon, Inc. (Lafayette, CO). Briefly, a CRISPR mixture containing gRNA and 20 ng/μl eSPCas9 protein (Cat # ESPCAS9PRO-50UG) (Millipore-Sigma, Burlington, MA, USA) in injection buffer (0.1 mM EDTA, 10 mM Tris-HCl, 100 mM NaCl) was introduced into WT C57BL/6J fertilized eggs via pronuclear microinjection following standard methods. Guide RNA efficacy and the status of non-homologous end joining (NHEJ) were validated by PCR genotyping of blastocysts in the initial phase and subsequently on tissue samples isolated from transgenic founders and progeny. Deletion mutations were identified by genotyping and sequencing, and founder 3 (male) was chosen to generate the knockout line. The knockout mice were backcrossed to WT C57BL/6 mice for 2 generations before generating the homozygous mice. WT C57BL/6 mice were used as controls and were purchased from The Jackson Laboratory (Bar Harbor, ME) and bred in-house at Cedars-Sinai Medical Center.

### IFNα2A^-/-^ mice genotyping

The following primers were used to screen IFNα2A^-/-^ mice: IFNa2A-F: TCAACCTCTGCAAGACCCAC and IFNa2A-R2: GAGTCTAGGAGGGTTGTATTCC. The resulted PCR product is 181 bp for deletion mutant allele and 341 bp for wild type allele.

### Ocular infection

WT control and IFNα2A^-/-^ mice (7 to 8 weeks old of both sexes) were ocularly infected in both eyes with 2 X 10^5^ pfu/eye of HSV strain McKrae in MEM medium as an eye drop without corneal scarification as we described previously [61].

### Viral titers from tears of infected mice

Tear films were collected from 10 WT (5 males and 5 females) and 17 IFNa2A (10 males and 7 females) infected mice eyes on days 1–5 post infection (PI) using a Dacron-tipped swab. Each swab was placed in 1 ml of tissue culture medium and squeezed. The amount of virus was determined using a standard plaque assay on RS cells as described [105]. Virus replication in the eyes of infected male and female mice for each group were the same.

### Monitoring corneal scarring

The severity of CS lesions in mouse corneas was examined by slit-lamp biomicroscopy. Scoring scale was: 0, normal cornea; 1, mild haze; 2, moderate opacity; 3, severe corneal opacity but iris visible; 4, opaque and corneal ulcer; 5, corneal rupture and necrotizing keratitis as we described [106].

### *In vitro* explant reactivation assay

TG from infected mice were harvested 28 days PI, cultured in 1.5 ml tissue culture medium, and the reactivation assay was performed as we described previously [107]. Briefly, a 100-μl aliquot was collected from each culture daily and used to infect RS cell monolayers, which were monitored daily for 5 days for the appearance of cytopathic effect (CPE) to determine the time that reactivated virus first appeared from each TG. Media from the explanted TG cultures were plated daily and the time at which reactivated virus first appeared in explanted TG cultures was determined.

### Gene expressions during primary infection

TG from individual mice were isolated on days 3 and 5 PI. Collected tissues were immersed in TRIzol reagent and stored at −80°C until processing. TG from each animal were processed for RNA extraction as described previously [107]. Isolated total RNA was reverse transcribed using high capacity cDNA reverse transcription kit (Applied Biosystems, Foster City, CA) according to the manufacturer’s recommendations. Expression levels of gene transcripts were evaluated using custom-made TaqMan primer sets as follows: for gB, 5′-AACGCGA CGCACATCAAG-3′ (forward), 5′-CTGGTACGCGATCAGAAAGC-3′ (reverse), and probe 5’ FAM-CAG CCGCAGTACTACC-3′ (where FAM is 6-carboxyfluorescein); CD8α (Mm01182107_g1); IFNγ (Mm01168134_m1); IFNα1 (Mm03030145_gH); IFNκ (Mm02529417_s1); IFNα4 (Mm00833969_s1); IFNα6 (Mm01703458_s1); IFNα11 (Mm04207507_gH); IFNα12 (Mm00616656_s1); IFNα13 (Mm01731013_s1); IFNα14 (Mm01703465_s1); IFNα16 (Mm01703434_s1); Stat1 (Mm01257286_m1); Stat2 (Mm00490880_m1); CD11c (Mm00498701_m1); IRF9 (Mm00492679_m1); CD11b (Mm00434455_m1); NK1.1 (Mm07307455_s1); F4/80 (Mm00802529_m1); CD4 (Mm00442754_m1); and IFNβ (Mm00439552_s1). GAPDH (m999999.15_G1) was used as an internal control.

The estimated relative copy number of gB expression was calculated using standard curves generated from plasmid pAc-gB1 [108]. Briefly, plasmid DNA template was serially diluted 10-fold so that 1 μl contained from 10^3^ to 10^8^ copies of the desired gene and then was subjected to TaqMan PCR with the same set of primers as the test samples. The copy number for each reaction product was determined by comparing the normalized threshold cycle (CT) of each sample to the threshold cycle of the standard curve. To analyze fold change of expression, the 2−ΔΔCT method was used to calculate gene expression fold change compared to expression in uninfected controls in each group. Statistical difference was calculated by ΔCT value.

### Determining the levels of latency in TG of latently infected mice

TG from individual mice were collected on day 28 PI, immersed in RNA stabilization reagent (RNA Later, Thermo Fisher Scientific, Waltham, MA, USA), and stored at −80°C until processing. Total RNA was extracted as we described previously. LAT RNA levels from latent TG were determined using custom-made LAT primers and probe as follows: forward primer, 5′-GGGTGGGCTCGTGTTACAG-3′; reverse primer, 5′-GGACGGGTAAGTAA CAGAGTCTCTA-3′; and probe, 5′-FAM-ACACCAGCCCGTTC TTT-3′ (amplicon length = 81 bp). GAPDH was used as an internal control. LAT RNA copy number was calculated using a standard curve generated using pGem5317 containing the LAT region as we described previously [61].

### Statistical analysis

Student’s t-test was performed using the computer program Instat (GraphPad, San Diego, CA). Results were considered statistically significant at a “P” value of <0.05. All experiments were repeated at least two times to ensure accuracy. For RT-PCR analysis, the statistical significance was calculated based on ΔΔCT and P<0.05 considered as significant. Error bar for each figure was automatically calculated by PRISM software, which is based on individual value of fold change, 2^(- ΔΔCT).

## Supporting information

S1 Data Original data that underlies this paper (Figs 2–7). (PDF)
