# Supplementary material for: The importance of IFNα2A (Roferon-A) in HSV-1 latency and T cell exhaustion in ocularly infected mice
Source: PLoS Pathog. 2024 Oct 1;20(10):e1012612. doi: 10.1371/journal.ppat.1012612 (PMC11469491; doi:10.1371/journal.ppat.1012612)
Supplement: S1 Data — (PDF) [file ppat.1012612.s001.pdf]

Figure 2A

Day1			Day2			Day3			Day4			Day5	
WT	IFNa2		WT	IFNa2		WT	IFNa2		WT	IFNa2		WT	IFNa2
0	100		7650	300		3150	400		1000	200		240	510
2050	110		2050	5350		0	250		0	5100		600	700
0	210		200	4950		0	250		200	1150		360	100
0	560		1550	750		250	0		6500	350		1050	50
190	0		650	6100		9000	1200		4300	4450		850	0
0	1100		100	100		1000	800		1450	1100		320	710
10	0		0	1300		1900	150		6950	1400		250	1360
720	10		2000	2300		1450	1150		2400	3000		1260	2010
310	0		350	0		7100	0		700	1650		860	1540
200	310		300	0		0	0		1000	0		1140	350
410	30		0	6550		5050	5600		5250	2300		0	460
640	0		4550	7850		8100	3250		4150	150		480	180
30	10		0	2550		300	5700		1050	400		610	390
250	0		150	3700		2550	50		0	0		1920	160
230	1230		7800	1650		0	700		450	0		0	1640
2100	30		1100	1800		0	150		350	1550		0	660
10	790		5350	2700		6200	9000		450	6900		100	230
140	320		2250	850		300	100		0	1200		190	1290
820	40		2300	0		500	100		1600	5650		210	410
0	0		6750	0		100	200		0	1650		1330	1180
	250			5100			50			2250			0
	10			0			250			750			2090
	40			850			1100			0			150
	100			4200			1550			0			390
	0			650			0			50			1520
	60			4100			3400			400			1390
	510			0			0			1950			70
	900			0			450			0			10
	1340			2950			50			1300			100
	710			400			8900			850			0
	2100			950			1050			150			1040
	480			100			8100			300			0
	210			1300			0			0			810
	260			0			550			2850			470

Figure 2B

Day3			Day5	
WT	IFNα2		WT	IFNα2
2128628	246344.8		18568.97	4294.365
627793	1063482		9920.629	5005.181
5864515	1601927		20471.51	21662.69
6627883	1228635		602.1793	472.1872
9385585	7273088		25211.32	25482.36
2974305	463.8806		25539.38	28914.08
1728713	112758.6		3539.926	31235.06
	3662224		17909.7	2116.29
1003306	19380.36		12840.75	8381.335
530593.8	6836261		472.824	4.205526
711325.1	3652595		19202.22	28881.02
4362243	3010665		4991.127	1138.538
			11637.58	10911.82
			20345.01	46.92187
			13512.45	1068.46
			1195.417	14308.73
			29197.86	653.4996
			17247.67	6.413625
			1688.355	30.40637
			19801.17	1356.603
			8088.925	2.703626
			3772.886	1251.224

Figure 3a

	WT											
IFNα1	11.15114	0.180825	0.648359	5.376264	0.190739	0.159966	0.228602	0.122907	0.609303	0.249848	10.72571	9.881138
IFNκ	0.782711	0.964833	1.363843	1.233889	1.60627	1.144546	1.01534	0.9919	1.341027	1.543925	1.206078	0.569706
IFNα6	1.968634	0.544898	1.307706	0.824277	1.283834	0.063372	2.435792	0.742894	3.102907	0.625563	1.981782	1.609913
IFNα12	0.206456	0.954344	0.306734	1.301111	0.313578	0.78094	0.39906	0.178985	1.106515	0.898526	0.580693	0.437612
IFNα13	1.16333	0.061126	0.67603	2.224941	1.773075	1.649202	1.329857	0.990673	1.862263	0.543137	2.726781	2.745161
IFNα16	0.951369	0.608172	0.576995	1.923691	2.419741	0.790185	1.234995	1.007386	0.859511	2.325453	2.230379	4.770965
	IFNa2											
IFNα1	2.380659	0.24087	0.127643	0.474914	0.146717	0.128343	0.221892	1.958548	0.230383	0.269887	14.90643	3.466081
IFNκ	0.911303	0.978336	1.225536	1.238244	1.066166	1.468017	1.772363	2.860356	1.766248	1.88238	1.576349	1.991465
IFNα6	1.212491	1.19086	1.052104	1.092837	1.426866	2.67745	1.417679		1.733299	1.912653	2.463201	1.848519
IFNα12		0.322443		0.327197	0.436282	0.58191	0.161888	0.215277	0.348163		0.401379	1.539755
IFNα13	0.544643	1.330349	0.970644	0.294956	0.369965	0.77331	0.330398	1.260153	0.827291	0.530257	3.515145	2.406688
IFNα16	1.215649	0.501199	1.979346	1.160933	1.566829	0.43302	0.54041	0.617331	0.763249	1.916147	0.549401	0.837353

Figure 3B

	WT									
IFNα1	3.16556	0.720895	6.476398	4.395106	0.924444	7.165309	4.356872	1.455786	6.569326	2.251664
IFNκ	1.702249	2.298569	1.32082	1.641262	2.133973	1.71918	0.819041	1.486227	1.161725	1.003584
IFNα6	3.870089	1.268563	8.241292	2.159024	0.74678	4.313412	8.055277	1.538132	5.341314	3.922408
IFNα11	3.745466	1.282252	6.205305	4.328295	1.352346	6.110227	6.946625	2.50378	3.776239	2.596554
IFNα13	12.17971	2.138189	30.21893	17.24433	1.817751	5.842005	16.02577	1.632676	9.598497	15.37413
IFNα14	1.841388	0.939359	2.200328	0.816517	0.488163	0.390796	1.341884		0.844274	2.294946
IFNα16	1.422036	0.66861	3.488643	2.216415	0.522522	0.906863	2.590178	0.793755	2.353044	2.188924
	IFNa2									
IFNα1	0.88477	1.747023	0.551257	2.794536	0.927223	0.860099	0.195237	3.204264	0.614635	0.962137
IFNκ	3.72506	1.509272	2.261674	1.35566	0.757115	1.756636	2.307085	1.437706	1.619388	1.358474
IFNα6	0.141595	6.211894	1.583652	4.777915	0.854356	1.152773	0.005648	7.48995	0.823992	0.355377
IFNα11	0.99742	2.314283	3.051376	4.258671	1.304997	0.992758	0.707167	2.756749	0.307329	1.549076
IFNα13	1.967401	1.677556	64.99264	9.164129	54.43703	1.527123	1.155221	3.646801	2.136254	2.669251
IFNα14		0.783967		0.797591				0.860547	0.079233	1.484042
IFNα16	0.786587	0.810583	2.068761	0.57293	3.208565	0.60977	1.841318	0.634358	0.761423	1.034611

Figure 4A

	WT											
CD4	0.538814	0.658871	1.659176	0.782145	1.455364	2.066551	1.088609	1.95182	0.827016	0.988669	1.510195	1.228348
CD8α	1.204779	0.603984	1.788747	1.739844	1.772199	1.608869	0.91284	2.02539	0.847792	0.867436	5.826448	3.40101
F4/80	0.338232	1.024245	0.934589	0.775718	1.54703	1.415752	0.905444	1.241407	0.82525	0.969487	1.27717	0.727791
CD11b	0.941237	1.551767	0.671936	0.547092	3.73052	0.502443	0.924896	1.044746	1.143345	0.623101	0.515678	0.340186
CD11c	2.295705	4.467822	0.718277	0.322256	1.091751	0.471174	1.12056	1.000372	1.437333	1.489614	0.374546	0.564347
NK1.1	0.560522	1.046707	1.32892	1.698191	0.93196	1.313788	0.953422	1.178423	0.744268	1.705684	2.292025	2.039579
IRF9	3.628167	1.044649	2.331897	2.07913	1.242656	1.234646	0.950014	1.119655	1.014273	0.751259	4.336869	4.033214
Stat1	4.841888	0.737329	3.206001	2.83817	1.493547	1.263667	0.789459	0.758483	0.949787	0.893345	6.933087	8.908789
Stat2	5.463616	1.130401	2.563302	2.613627	1.39124	1.203163	0.924915	1.002556	0.952712	0.787782	5.725989	6.074306
	IFNa2											
CD4	1.874668	0.817716	1.05131	1.320713	1.535252	2.543821	2.571194	2.181481	1.222993	1.416692	0.811648	1.514481
CD8α	0.411982	0.875307	0.837804	1.070811	0.813467	1.240569	1.273596	2.681758	1.036148	1.741743	0.869274	3.614056
F4/80	0.736232	0.578171	1.118265	1.140384	1.419407	4.184958	1.294515	1.043093	0.851848	0.914129	1.357365	6.249182
CD11b	0.951727	0.904678	0.532387	1.432111	0.807503	0.65173	2.945817	2.390553	0.528398	5.202614	1.061996	6.412448
CD11c	0.904625	1.261714	0.940211	1.411815	1.276013	1.226881	4.652077	4.897206	0.53612	1.461408	1.897407	1.932276
NK1.1	0.950294	0.647631	1.213039	1.347263	1.171032	1.4581	1.109705	1.276822	0.778321	1.061903	2.374565	3.272077
IRF9	2.569702	1.037384	1.157522	0.788066	1.649592	1.370196	1.278008	2.941068	1.03392	0.960498	3.932319	3.184224
Stat1	4.107093	0.77162	1.083829	1.017652	2.510069	1.372936	1.296594	3.806039	0.967965	1.266908	9.721264	6.241375
Stat2	3.289342	1.037463	1.051039	0.834365	2.257176	1.244325	1.177178	3.163319	0.762123	1.187425	6.177873	4.080464

Figure 4B

	WT									
Stat1	13.05702	11.70181	12.84993	15.00841	14.40801	15.72911	12.2686	7.99561	13.40117	10.03846
Stat2	9.958686	7.652276	11.44656	12.56565	11.28161	12.85872	10.08422	5.750194	10.2635	7.191968
F4/80	3.279727	1.785545	5.641167	5.204838	1.161749	1.677317	2.91734	1.687835	4.232918	4.023723
NK1.1	16.26166	6.686006	25.58492	20.87249	5.618764	24.68832	10.69502	7.005676	20.13554	12.41156
IRF9	5.167671	4.443256	5.126552	6.362264	6.351185	8.041731	5.137079	3.905323	6.130561	4.539161
CD11b	8.393494	2.635061	12.50153	5.977469	1.60271	3.408515	8.754254	2.425734	8.306678	11.37661
CD4	3.892887	1.137176	3.911377	2.401256	0.64933	3.115736	2.24087	2.014404	4.553297	3.885322
CD11c	14.65809	4.594227	23.38757	15.51581	3.066966	5.407486	12.90483	4.397982	17.86109	19.21995
CD8a	29.95588	8.369364	29.35505	18.78423	5.328887	27.07149	18.01188	10.64618	25.29038	20.79201
IL10	170.9191	47.40282	160.832	159.0808	45.9108	315.8844	90.13546	95.64616	294.9325	127.4356
GZMA	310.2338	89.30447	603.9268	496.0754	85.3315	405.0921	214.2775	133.3715	431.3956	271.045
	IFNa2									
Stat1	0.914701	14.2027	2.030459	12.2887	2.524328	0.869744	1.186168	12.90219	0.798958	11.03599
Stat2	1.336802	11.82215	1.997295	12.35407	2.053508	1.094543	1.53789	10.90928	1.086466	9.418786
F4/80	1.532869	2.2361	1.010454	3.794537	0.74348	0.912382	1.45986	1.829541	1.668568	1.438912
NK1.1	2.053917	33.51157	1.620894	16.35543	1.938983	0.65284	2.192432	20.16075	0.919926	6.738957
IRF9	1.448237	6.88161	3.999457	6.457343	3.485342	1.020113	1.875508	5.911732	1.103775	5.456369
CD11b	1.481794	6.202566	4.948215	10.34841	4.532087	0.587012	1.325326	6.678734	0.639299	3.725066
CD4	1.113314	7.515642	0.791995	5.315543	0.766261	0.457337	0.633162	4.441556	0.567562	1.860958
CD11c	1.202112	10.36659	1.334644	16.3428	1.561724	0.423167	0.790262	13.30037	0.435831	5.48005
CD8a	1.379958	42.41335	0.64375	24.65827	1.634524	0.821646	0.825244	32.94514	0.494957	8.539481
IL10	2.408845	324.19	4.895334	255.1248	9.856726	1.3531	1.888303	209.2423	0.644866	71.0211
GZMA	1.652443	582.4188	6.09266	460.5602	15.46767	1.256789	4.519704	437.0269	3.178722	125.7438

Figure 5A

	WT											
IFNγ	55.08199	0.678647	15.75216	47.09819	0.953044	1.935831	0.362948	0.875292	0.943977	0.42915	40.83915	86.64357
IFNβ	27.77883	26.73577	0.316661	0.37155	0.358266	0.140158	0.218386	0.530231	8.607812	2.83806		14.90053
	IFNa2											
IFNγ	5.473977	1.11954	0.808458	0.799395	1.026482	1.654873	1.9839	41.90697	0.943907	1.399341	54.14757	55.61938
IFNβ	0.231538	0.50571	0.623084	0.515001	0.431923	9.330468	8.888566	29.51369		0.467169	3.556619	1.693745

Figure 5B

	WT									
IFNβ	16.36488	1.051118	51.66984	25.9072	2.884499	22.47487	57.82922	2.809483	47.47279	28.74915
IFNγ	90.3114	387.9861	863.8304	205.9327	605.5867	761.6512	594.7241	105.0346	1140.022	773.6356
	IFNa2									
IFNβ	0.234262	3.916552	0.326088	24.0333	1.747855	0.365818	0.315176	6.80053	0.185673	9.056752
IFNγ	0.757621	682.7184	7.329975	1136.358	19.94059	0.979932	3.386691	640.12	3.373621	321.7995

Figure6A

WT	IFNα2-/-
46005212	21680520
19564796	31154858
69382724	55401795
49482545	62435408
59787103	66600982
46767620	16757434
33227915	31293598
97363590	3038688
62776186	47152177
45088563	6470508
28993322	75010176
83667800	79201135
56651133	58882435
23938546	51974623
52587684	34595373
26534439	8123998
46820287	43536028
1.17E+08	78758452
1.07E+08	4100704
75151113	9666521
99144893	14421751
348797.9	83862137
72354335	41743029
71098036	638363
24066175	81691588
91300825	1.2E+08
38910000	86292687
67008515	19912423
33369348	1.71E+08
1.68E+08	1.07E+08
1.57E+08	11865.99
1.24E+08	2.25E+08
1.01E+08	1.12E+08
4.33E+08	1.62E+08
1.62E+08	51355544
2.69E+08	55611998
1.17E+08	29873747
3.69E+08	43294969
3.72E+08	94551001
2.55E+08	35232345
	67898759
	75946166

Figure 6B

WT	IFNα2$^{-/-}$
3	3
3	
3	4
4	3
4	3
	4
3	4
3	4
4	4
3	3
3	3
3	
7	4
3	4
4	3
4	
3	
4	
3	

Figure 6C

WT	IFNα2$^{-/-}$
0	1
0	0
0	0
2	0
0	0
0	0
3.5	0
0	0
0	0
0	0
0	3.5
0	0
0	0
0	2
2	0
0	0
0	0
0	3.5
0.5	2
0	0
0	0
3.5	0
0	0
3.5	0
0	0
0	0
4	0
0	0
3	0
3.5	0
0	0
0	0
1.5	0
4	3.5
0	0
0	0
0	1
0	0
0	
0.5	
3	
0	
0	
0	
2	
0	

Figure 7A

	WT			IFNα2$^{-/-}$		
	mean	sem	n	mean	sem	n
CD8α	21.38164	3.945072	22	12.08811	2.289626	35

Figure 7B

	WT			IFNα2$^{-/-}$		
	mean	sem	n	mean	sem	n
PD-1	16.54514	3.812511	22	9.1196	1.793228	35
